# Occupational Exposures in Commercial Laundry and Dry Cleaning Industries and their Associative Cancers: A Scoping Review

**DOI:** 10.21203/rs.3.rs-3250169/v1

**Published:** 2023-09-13

**Authors:** Emma Landskroner, Candace Su-Jung Tsai

**Affiliations:** University of California, Los Angeles; University of California, Los Angeles

**Keywords:** Cancer, dry cleaning industry, hazardous chemical exposure, indoor exposures, occupational exposures, solvents

## Abstract

**Background:**

The laundry and dry cleaning industries are critical for maintaining cleanliness and hygiene in our daily lives. However, these industries have also been identified as sources of hazardous chemical exposure for workers, leading to potentially severe health implications. Despite mounting evidence that solvents like perchloroethylene and trichloroethylene are carcinogenic to humans, they remain the most commonly used solvents in the industry. In addition, while alternative solvents are increasingly being utilized in response to evidence of adverse health and environmental effects, there remains a significant gap in our understanding of the potential risks associated with exposure to these new agents.

**Methods:**

A systematic scoping review was conducted to identify prevalent toxic substances in the commercial laundry and dry cleaning industries that workers are exposed to and, further, to identify gaps in the existing literature regarding those exposures and related cancer development. Reported study exposure values were compared with current occupational exposure limits and biological exposure indices.

**Results:**

Most studies examined perchloroethylene exposure in the dry cleaning industry, with one notable finding being that genotoxic effects were found even below current occupational exposure limits. Separate studies on TCE and benzene presented varied exposure levels and health risks, raising concern due to their IARC Group 1 carcinogen classification. Lastly, data on alternative solvents was limited, with a lack of health outcome data and gaps in their exposure guidelines and carcinogenic classifications.

**Conclusion:**

A gap in research exists regarding health outcomes, particularly cancer development, from solvent exposure in the dry cleaning industry. Most studies (66%) overlooked health implications, especially for emerging solvents. Further, results showed potential DNA damage from the established solvent, perchloroethylene, even below current occupational exposure limits, emphasizing the need to reevaluate safety limits. As alternative solvents like butylal and high-flashpoint hydrocarbons become more prevalent, investigations into the effects of their exposure are necessary to safeguard workers’ health. This scoping review is registered with the Open Science Framework, registration DOI: https://doi.org/10.17605/OSF.IO/Q8FR3

## 1. Background

The laundry and dry cleaning industry in the United States comprises approximately 36,000 facilities employing 157,400 workers (1). These facilities commonly use solvents and hazardous chemicals for deep cleaning and stain removal (2, 3, 4). The International Labour Organization (ILO) reports that inhalation of solvents is the most common method of occupational exposure, with acute, high levels leading to delirium, respiratory depression, and death, and chronic low levels being associated with cancer, reproductive issues, and neurotoxicity (4, 5, 6).

The pollutants generated from these operations have been linked to harmful environmental impacts, including air pollution and groundwater contamination, a potential secondary route of exposure (4, 5, 6). While the health impacts of acute solvent exposure in the laundry and dry cleaning industry are relatively consistent across solvent types, the effects of chronic exposure vary depending on the specific solvent involved (4). Given the diverse range of solvents with intrinsic differences utilized in the industry, paired with the continuous development of new, green alternative types, this has led to an area of research that needs further exploration.

Current evidence suggests that dry cleaning and laundry workers are at an increased risk of cancer mortality (7, 8). In a National Institute for Occupational Safety and Health (NIOSH) study by Ruder et al. involving 1,708 dry-cleaning workers exposed to common solvents like perchloroethylene (PCE) before 1960 and followed until 1996, a significant excess of total cancer deaths was observed (271 deaths, Standardized Mortality Ratio (SMR) 1.25, 95% Confidence Interval (CI) (1.11–1.41)) (7). Further analysis revealed statistically significant SMR results for tongue, bladder, esophagus, intestine, lung, and cervix cancer, with tongue cancer and ischemic heart disease elevated among individuals solely exposed to PCE (7).

Supplementary, a study by Callahan et al. conducted an extended mortality follow-up among dry cleaners and found a significant exposure-response relationship for bladder cancer and kidney cancer, as well as a correlation between chronic exposure and heart disease and lymphatic/hematopic malignancies (9). Carton et al. and Vlaanderen et al. also assessed occupational exposures in relation to cancer development. Dry cleaners were among the most frequently exposed occupation, with increased odds ratios for squamous cell carcinoma of the head and neck, particularly with exposure to PCE and trichloroethylene (TCE) (10). Chronic exposure to PCE was associated with elevated hazard ratios for liver cancer, non-Hodgkin’s lymphoma, and multiple myeloma; however, no association was observed between TCE exposure and any of the identified cancers (11).

Although there are available publications examining solvent exposure in the dry cleaning industry, the vast majority primarily focus on PCE, the most popular solvent type (12, 13). Despite compelling evidence of its adverse health outcomes, and efforts to regulate and reduce its prevalence, PCE remains the industry standard (12, 13). In response to growing concern over the use of harmful solvents, the industry is exploring new and more ecologically sound options with unknown health implications for workers. As a result, individuals in the dry cleaning industry remain at risk of exposure to already established hazardous substances, like PCE and TCE, and exposure to emerging solvents with unknown health impacts.

### 1.1. Perchloroethylene and Trichloroethylene

TCE and PCE, common solvents classified as volatile organic compounds (VOCs) (defined in Supporting Information (SI) VOC Section), are widely used chemical solvents in the dry cleaning industry (5). These non-flammable and colorless solvents are effective stain removers and are commonly used in the US and Europe (11, 12). Based on extensive epidemiological evidence, the International Agency for Research on Cancer (IARC) has classified PCE as probably carcinogenic to humans (Group 2A) and TCE as definitively carcinogenic to humans (Group 1), linking exposure to increased cancer risks (5, 12). PCE exposure is associated with liver, kidney, and central nervous system damage, as well as an elevated risk of bladder cancer and non-Hodgkin lymphoma (5, 14, 15, 16). Comparatively, there’s substantial human evidence demonstrating a positive association between TCE exposure and kidney cancer, as well as an increased risk of non-Hodgkin’s lymphoma, cervical cancer, and liver cancer, while in vivo data indicates TCE induces tumor development in the liver, lungs, testes, and hematopoietic tissue (5, 17, 18, 19, 20).

Occupational exposure limits (OEL) for PCE and TCE vary across countries and agencies. In the United States, the Occupational Safety and Health Administration (OSHA) has set a permissible exposure limit (PEL) for both PCE and TCE at 100 parts per million (ppm) as an 8-hour time-weighted average (TWA) (21, 22). The American Conference of Governmental Industrial Hygienists (ACGIH) establishes a threshold limit value (TLV) of 25 ppm for PCE based on an 8-hour TWA, with a short-term exposure limit (STEL) of 100 ppm for 15 minutes (23). Regarding TCE, the ACGIH sets the TLV-TWA at 10 ppm and the TLV-STEL at 25 ppm (24). While NIOSH lacks a specific quantitative recommended exposure limit (REL) for PCE, advising the lowest feasible concentration, it does provide a REL for TCE at 25 ppm based on a 10-hour TWA (25, 26).

Occupational exposure to these solvents is a significant concern for laundry and dry cleaning workers, as prolonged exposure to PCE and TCE can lead to serious health problems (27). Given the prevalence of these solvents in the industry, workers are at a high risk of exposure.

## 2. Alternatives to PCE and TCE

As increasing evidence is published substantiating PCE and TCE’s negative health and environmental impacts, regulatory agencies worldwide have taken steps to phase out or restrict their use (28). For example, the United States Environmental Protection Agency (EPA), the European Union’s Registration, Evaluation, Authorization, and Restriction of Chemicals (REACH) regulations, and Australia’s National Industrial Chemicals Notification and Assessment Scheme (NICNAS) have implemented guidelines to limit these solvents in the dry cleaning industry (29, 30, 31, 32, 33, 34, 35). Their rules aim to promote the adoption of safer solvents and alternative cleaning methods. The Toxic Use Reduction Institute (TURI) assessed alternative options to PCE in dry cleaning, ranking them based on technical, economic, environmental, regulatory, and health factors (36). The alternatives were ranked from one to five, with one being the most desirable: 1. Wet cleaning (water and detergent without solvents); 2. Liquid carbon dioxide (used with specialized detergents under the pressure of 700 PSI); 3. High flashpoint hydrocarbons, propylene glycol ethers, and butylal; 4. Siloxane; 5. N-propyl bromide (12, 36).

Among the five substitution options, the two safest alternatives, wet cleaning, and liquid carbon dioxide, are non-solvent based. However, the adoption of these methods has been slow. Wet cleaning is not a complete replacement for solvent-based cleaning, is associated with a higher risk of fabric deterioration, shrinkage, difficulty removing specific stains, and is labor intensive (12). While liquid carbon dioxide entails the purchase of costly specialized machinery, presents safety hazards due to high-pressure systems, and lacks effectiveness in removing protein-based stains (37). Consequently, solvent-based methods remain the most prevalent approach for cleaning (12). Limited data is available on the health effects of the suggested alternative solvents, discussed in the following subsections, with the majority of alternatives yet to be examined and classified by the IARC, posing potential carcinogenic risks to dry cleaning workers.

### 2.1. High-Flashpoint Hydrocarbons

High-flashpoint hydrocarbons, ranked Group 3 by TURI, are made up of aliphatic hydrocarbons and are volatile petroleum-based solvents with a flashpoint at or above 140°F (60°C) (36, 38, 39). These solvents offer a better alternative to PCE and TCE, as they are generally less volatile and are not designated hazardous air pollutants (HAPs) or ozone-depleting substances (ODS), making them safer to handle and store (40, 41). As a result, they have become the most widely used alternative (38, 39).

High-flashpoint hydrocarbon solvents are manufactured under several trade names, two of the most popular being DF2000^™^ (ExxonMobil Corporation) and EcoSolv^®^ (Cheveron Phillips Chemical Company, LLC) (39). While these solvents have gained popularity in the United States, being touted as a greener alternative to PCE, there is still limited evidence on the potential carcinogenicity of exposure and no OEL standards (14, 39).

### 2.2. Propylene Glycol Ethers (PGE)

Propylene Glycol Ether (PGE) solvents, ranked Group 3 by TURI, are organic, volatile, water-soluble compounds with a flash point ranging from 160–212°F (71–100°C ) (36, 42). There are numerous types of PGE solvents available for dry cleaning; some of the most well-known formulations are glycol n-butyl (DPnB) and dipropylene glycol tert-butyl (DPtB), which have emerged as promising substitutes for PCE and TCE (43, 44). While certain PGEs are carcinogenic, DPnB and DPtB have not been linked to any adverse environmental or health impacts (43, 44). These biodegradable solvents are characterized by their low volatility and efficient cleaning properties (12, 43, 44).

OELs for glycol ether solvents vary depending on their specific compound. As for DPnB and DPtB, OSHA has set a legal airborne PEL for DPnB at 50 ppm and for DPtB at 100 ppm, averaged over an 8-hour work shift (45, 46). ACGIH has set a TLV of 20 ppm for DPnB, and 100 ppm for DPtB, averaged over an 8-hour work shift (45, 46). Lastly, NIOSH has set a REL for DPnB at 5 ppm and 100 ppm for DPtB, averaged over a 10-hour work shift (45, 46). While certain PGE solvents have established OELs, the IARC has not classified PGEs for carcinogenicity (36).

### 2.3. Butylal

Butylal, known as dibutoxymethane and ranked Group 3 by TURI, is a combustible liquid with a flash point of 144°F (62°C), is commonly used in the dry cleaning industry, primarily as SolvonK4 ^™^ (Kreussler Inc.). It contains butylal (> 99% purity), along with small amounts of n-butanol and formaldehyde (28, 36, 47). Limited data is available on the health effects of butylal, with most studies focusing on dermal and oral exposures. No OELs have been established for butylal, and the IARC has not reviewed its carcinogenicity (28, 36).

### 2.4. Siloxane

Decamethylcyclopentasiloxane, or D5, Group 4 by TURI, is colorless and odorless volatile methyl siloxane used as a solvent in the GreenEarth^®^ dry cleaning system (12, 36). Made of a combustible modified liquid silicone with a flashpoint of 170°F (76.6°C), D5 is a less aggressive cleaner than PCE. It has been identified as a more environmentally friendly alternative by the California Office of Environmental Health Hazard Assessment (36, 48). The IARC has not classified D5, as there is insufficient data collected examining its toxicity (48). There are currently no established OELs for D5 (36).

### 2.5. n-Propyl Bromide

N-Propyl Bromide (n-PB), or 1-bromopropane, Group 5 by TURI, is a volatile chemical similar to PCE and other halogenated hydrocarbon solvents, with differing flash points depending on the testing method (36). It was promoted as an alternative to PCE in the EU via REACH and later determined to be a “regrettable substitution” defined as “the substitution of hazardous substances with similar chemical structure and similar hazard properties or with substances with other effects of similar concern” (49). The United States EPA has since added n-PB to the Clean Air Act list of hazardous pollutants, as exposure causes irritation, neurologic effects, and possible damage to the nervous system (36, 50, 51).

OSHA and NIOSH do not have a PEL or REL-TWA listed for n-PB. However, ACGIH has set a TLV based on an 8-hour TWA at 0.1 ppm, and the California Division of OSHA has developed a PEL based on an 8-hour TWA at 5.0 ppm. Further, the IARC has classified n-PB as possibly carcinogenic to humans (Group 2B) (50, 52, 53).

## 3. Rationale

Exposure to hazardous chemicals, such as volatile organic compounds, solvents, and other agents in the laundry and dry cleaning industry, threatens human health. Despite their widespread use, there is still a limited understanding of the specific cancers and chronic diseases that may be associated with these substances. Consequently, a scoping review was determined to be the most appropriate method to address this gap in the literature and provide a comprehensive understanding of the potential health risks linked to occupational exposure in this industry.

## 4.1. Methods

The scoping review was conducted in accordance with the Joanna Briggs Institute methodology for scoping reviews (54).The primary and secondary research questions guiding this review aimed to identify toxic substances in the laundry and dry cleaning industry and their potential links to cancer. Studies of various designs were included and charted in tables to ensure a thorough analysis. A Reporting Items for Systematic Reviews checklist - extension for Scoping Reviews (PRISMA-ScR) was used to guide the steps followed in this scoping review (55).

## 4.2. Search Strategy

The search strategy was designed to identify relevant peer-reviewed articles related to occupational exposures in the laundry and dry cleaning industry and any associated cancers. To achieve this objective, an initial limited search was performed on several reputable databases. The search terms used were based on the words and phrases found in the titles and abstracts of relevant articles, which were then used to formulate a comprehensive search strategy. This process ensured that all relevant studies were identified and included in the review.

Search terms were developed based on three primary categories: Occupational-related terms, exposures of interest-related terms, and one outcome of interest term (SI Table S2). A general search algorithm was developed and adapted for each included database. A systematic search of peer-reviewed literature published in English between January 1, 2012, and December 1, 2022, was performed via PubMed, Science Direct, NIH Library, Embase, EBSCOhost, and Google Scholar.

The population of interest comprised laundry and dry cleaning workers. The exposure scope was limited to relevant and commonly used chemicals throughout the dry cleaning process. The outcome of interest was exposure concentration of airborne chemicals and any information available regarding the potential adverse health outcomes, specifically cancer development due to exposure. Relevant studies of various designs, such as risk assessments, cohort studies, case studies, and biomonitoring studies, were included in the search strategy. Overview articles, commentary, editorial, or opinion articles were excluded to ensure the reliability and quality of the review’s findings.

## 4.3. Screening of Articles

**Figure 1.**
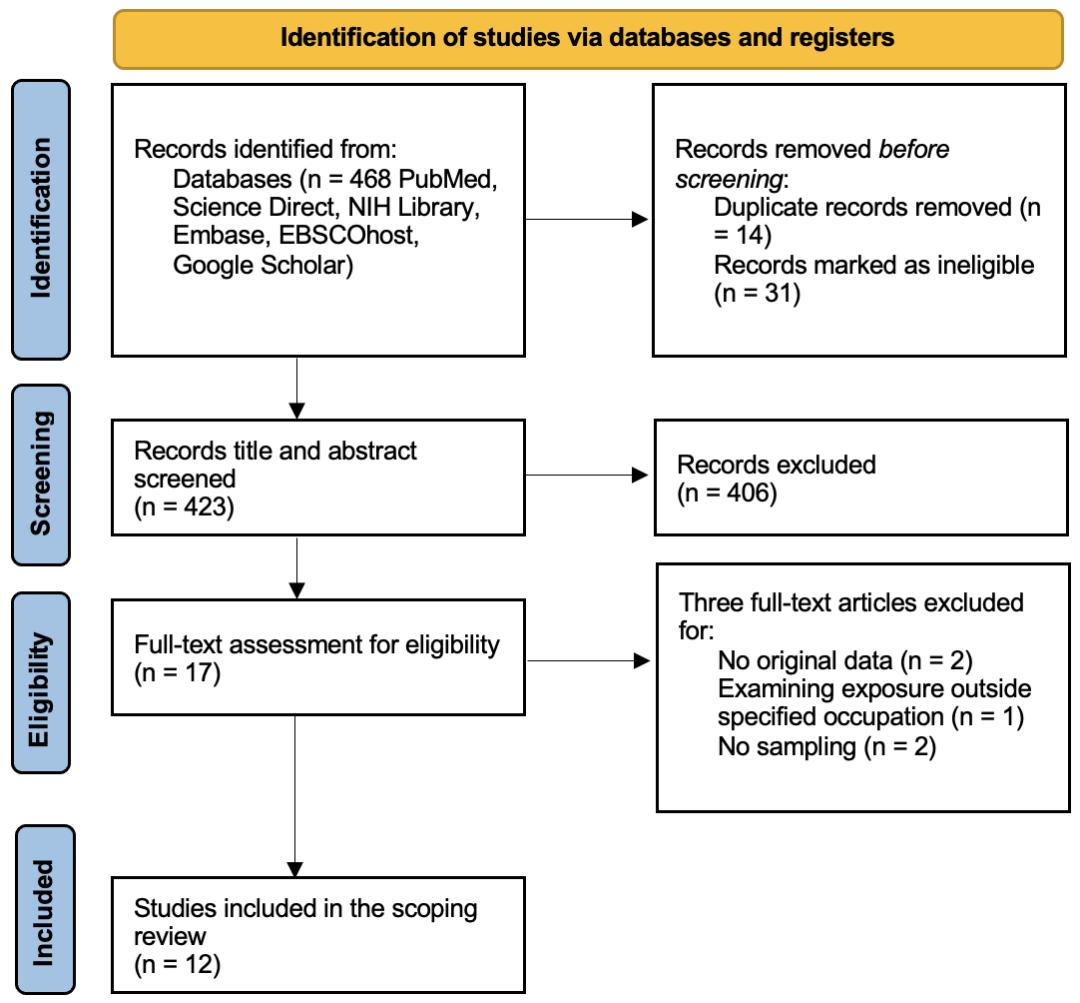
PRISMA Flow Diagram for the screening and selection of articles.

Articles were retrieved from the first systematic search and uploaded into EndNote 20.5 (SI Table S3). Duplicate articles and ineligible articles were identified and removed. The remaining articles’ titles and abstracts were independently screened against the specified inclusion and exclusion criteria (SI Table S1). Articles that did not meet the criteria were excluded. All articles that did meet the requirements were pulled for a full-text review. The remaining articles were approved and included in the scoping review (SI Table S4). The discussed screening and selection process is presented via Version 1 PRISMAs Flow Diagram (Fig. 1).

## 4.4. Data Extraction

The primary author (EL) extracted data from the selected articles. The data extracted included the first authors’ names, country of origin, the number of workplaces, the total number of workers, exposure measurement conditions, exposure agents of interest, measured outcomes, and reported health impacts if specified (i.e., cancer). The indication “NA” was marked if data was unavailable. In studies where sampling was conducted, outcomes were grouped by sampling type, i.e., Area Air Sampling, Personal Air Sampling, and Biological Sampling. When appropriate, subcategories were used to indicate when multiple measurements and sampling methods were used within a single study.

Data on area air or personal air concentrations in ppm used in some studies were converted to mg/m^³^ as follows (56):

$${\text{Concentration}\;\text{mg}/\text{m}}^{3}\;=\;\left(\text{ppm}\right)\;\times \;\left(\text{molecular}\;\text{weight}\right)\;\times \;\left(1/24.45*\right)$$

*Molar volume of gas at 1 atmosphere and 25°C (unless another temperature was specified in the study, noted in charting).

Data on airborne concentration found in the included studies were compared with current OELs from OSHA, ACGIH, and NIOSH. Data on biological concentration found in included studies were compared with current Biological Exposure Indices (BEI) set by the ACGIH. “NA” was used to designate when no OEL or BEI was available for an examined substance.

## 5. Results

### 5.1. Selected Studies

A PRISMA flow chart (Fig. 1) summarizes the screening and selection process. From an initial pool of 468 articles, 12 were identified for the scoping review after eliminating duplicates (n = 14), ineligible articles (n = 31), and articles that did not fit the specified criteria (n = 411). The 12 included studies were conducted in ten countries across the globe, four reporting area sampling only (n = 4: 1 Various VOCs (Nonane, Decane, Undecane, Nonanal, Decanal, O-xylene, and Toluene); 1 TCE; 1 PCE; 1 TCE and PCE), one reporting personal and area sampling (n = 1: 1 butylal and high-flashpoint hydrocarbons (Df-2000)), four reporting personal and biological sampling (n = 4: 3 PCE; 1 PCE and trichloroacetic acid (TCA)), and three reporting biological sampling only (n = 3: 2 PCE; 1 benzene). Of the studies measuring air concentrations, the duration of measurements ranged from 15 min. to 8 hours, with one study (Sadeghi et al.) only specifying that samples were taken every 15 days without indication of the sampling time and another study not including the sampling methodology (Friesen et al.).

Data on ambient area air concentrations measured in the working environment are collected and presented in Table 1, where area air sampling can be defined as the measurement of indoor static air pollution at a fixed location of interest. It is important to note that area air sampling provides an overview of pollutants in the workplace and helps identify exposure hazards; however, it is insufficient for measuring induvial worker exposure (57). Accurate assessment requires personal air sampling within the worker’s breathing zone (58). Additionally, biomonitoring can be conducted to assess exposure to chemicals via the internal dose (59). These measurements can then be compared with the relevant OELs to evaluate exposure risk.

Of the five ambient air sampling studies, three presented specific measurements exceeding at least one OEL standard (Friesen et al., Habib et al., and Sadeghi et al.)

While Ceballos et al. measured butylal and high-flashpoint hydrocarbons, substances with no OELs to compare, and Eun et al. presented six chemicals, three of which (nonane, o-xylene, and toluene) having published OELs, all within OEL. Based on the extracted data, the air concentration measurements present a lack of consistency when compared to the OELs, as some measures exceeded OEL values, raising concern about the potential health risks faced by workers, while others fell within standards.

Data on personal air concentrations are collected in Table 2, where personal air sampling can be defined as measurements taken within the breathing zone of an individual (58). Of the five personal air sampling studies, all studies with available OELs to compare had measurements under OEL standards, except Lucas et al., reporting a maximum range value for PCE above ACGIH TLV-TWA standards. Caballos et al. also took personal air samples for butylal and high-flashpoint hydrocarbon; however, no OELs are available for those substances. The extracted data shows that measured concentrations in workers’ breathing zones were below the recommended OELs, indicating proper implementation of strategies to limit worker exposure.

Data on biological concentrations are collected in Table 3, where sampling can be defined as “a collection of human specimens and associated data for research purposes” (60). Of the seven studies that extracted biological samples, three measured exhaled air concentrations (Modenese et al., Dias et al., and Ziener & Braunsdorf), of which all measurements, except a PCE maximum range value (Modenese et al.), measured lower than ACGIH BEI for exhaled air. Four studies took blood samples (Lucas et al., Everatt et al., Azimi et al., and Shim et al.); one measured PCE concentration (Lucas et al.) with samples within ACGIH BEI. Two studies (Azimi et al. and Everatt et al.) conducted comet assay on peripheral blood lymphocyte samples to assess genotoxicity of PCE exposure, with Azimi et al. finding a significantly higher median tail length (TL), %DNA in the tail, and tail moment (TM) amongst the PCE-exposed group compared to the controls (TL: 25.85μm vs. 5.61μm; %DNA in tail: 23.03 vs. 8.77; TM: 7.07 vs. 1.03), but found no correlation between the duration of employment and DNA damage (61). Everatt et al. similarly saw an increase in mean TL among dry cleaners (10.45 vs. 5.77, P < 0.05), additionally observing a significant association between chromosome aberration (CA) frequency, employment duration, and frequency of exposure, as well as increased micronuclei (MN) and DNA damage in workers compared to controls (CA:1.04 vs. 0.59, P = 0.005; MN: 11.36 vs. 6.96, P < 0.05; DNA: 10.45 vs. 5.77, P = 0.05), however, found no difference in CA frequency between dry cleaners and controls (62). Shim et al. reported laboratory findings from two patients exposed to benzene, a Group 1 carcinogen, revealing evidence of liver damage and toxicity. Results supported the association between long-term benzene exposure and adverse effects on the human liver (63, 64). Lastly, two studies (Modenese et al. and Shim et al.) collected urine samples for benzene and trichloroacetic acid (TCA), respectively. Measurements in each study fell below the established ACGIH BEIs for concentrations in urine, potentially because the substances had already been excreted before sampling.

Table 4 presents all studies organized according to the type of substance analyzed, study design, relevant reported health impacts discussed in the paper, and the available corresponding IARC classifications. The findings reveal a concerning trend, as only 33% of the papers monitored associative health outcomes, of which 75% focused on PCE, an already classified Group 2A substance. This highlights a lack of modern research on health outcomes associated with alternative solvents. In addition, several substances lack IARC classification, including butylal, high-flashpoint hydrocarbons, nonane, decane, and undecane, while xylenes and toluene are classified as Group 3, indicating a lack of carcinogenetic research.

### 5.2. Investigated Chemicals

#### 5.2.1. PCE

PCE concentration was measured in eight studies across seven countries (UAE, Iran, Lithuania, Brazil, France, Italy, and Germany), with approximately 90 dry cleaning or laundry shops examined. One biological sampling study (Azimi et al.) did not report the number of shops included. Overall, 202 exposed dry cleaners were reported, with two area air sampling studies (Habib et al. & Sadeghi et al.) not reporting the number of individuals working in the laundry facility at the time of measurement. Data regarding the reported PCE concentrations and study characteristics are presented in Tables 1, 2, and 3.

Of the two area air sampling studies examining PCE, Habib et al. took measurements at four different facilities in three varying working positions: “(i) extracting PCE solvent from the drum to fill the dry-cleaning machine,” “(ii) unloading the clothes from the dry-cleaning machine into the tray,” “(iii) preparing clothes for steam press” (65). PCE concentration ranged from 1.15 –510ppm (7.798–3,458 mg/m^³^) (65). Position two, involving the unloading of clothing, demonstrated the highest maximum concentration of PCE exposure across three out of the four monitored dry cleaning shops (Facility A: 200ppm (1,356 mg/m^³^); Facility B: 63ppm (427.2 mg/m^³^); Facility C: 510ppm (3,458 mg/m^³^); Facility D:240ppm (1,627 mg/m^³^)) (65). With maximum concentration measurements at facilities A, C, and D exceeding OSHAs PEL-TWA of 100ppm (300 mg/m^³^) and all facilities exceeding ACGIHs TLV-TWA of 25ppm (169.5 mg/m^³^). Most samples taken at position two exceeded OEL standards, posing a potential threat to the workers’ health; however, the study did not measure and report on associated health outcomes (21, 23, 25, 65).

In the other area air sampling study conducted by Sadeghi et al., ten dry cleaning shops were assessed, with supplemental sampling collected from a gas station, underground soil, and effluent (66). Results of air sampling collected from the dry cleaning shops found the mean value for PCE levels in air samples to range from 42.7–516 μg/L (42.7–516 mg/m^³^), with a grand mean of 110.9 μg/L (110.9 mg/m^³^), and the maximum level measured being 960 μg/L (960 mg/m^³^) (66). Of the ten shops, only one, Facility Three, had a calculated mean (516 μg/L or 516 mg/m^³^) and maximum (960 μg/L or 960 mg/m^³^) exceeding OSHAs PEL-TWA and ACGIHs TLV-TWA (66). Facility Three’s PCE concentration range was noted to be 320–960 μg/L (320–960 mg/m^³^), outside of the referenced OEL. The study did not measure associated health outcomes among workers.

Six studies collected biological samples measuring PCE concentration. Four of those six studies also measured PCE concentration in personal air samples. Everatt et al. collected personal air and peripheral blood sampling from 59 volunteers (30 exposed dry cleaning workers and 29 controls) (62). Personal air samples were collected from all dry cleaners on two consecutive 8-hour shift workdays, and 10 ml of whole venous blood was taken on the first day of air sampling (62). Each subject had four lymphocyte cultures prepped within 2–4 hours after blood collection, which were then assigned for CA assay, MN assay, and comet assay (62). The mean PCE concentrations in personal air samples were 31.40 mg/m^³^ and ranged from 0–77 mg/m^³^, within the established OSHA and ACGIH OELs (62). Regarding the biological samples collected and examined for genotoxic effect, dry cleaners had higher MN frequency (MN/1000 binucleated cells) and DNA damage, measured by comet tail length compared to the control group (62). No significant relationship was observed between these effects and the level of PCE exposure sampled. However, the differences between these groups were significant, indicating that levels below the established OELs could potentially still cause genotoxic damage to the body (62). Furthermore, after stratification, the data also showed that longer employment duration and a greater frequency of exposure to PCE (> five days per week) were associated with a higher extent of CA (62).

Lucas et al. collected personal air and peripheral blood sampling from 50 exposed employees from 22 dry cleaning shops and were compared to 95 non-exposed individuals (67). Personal air sampling was performed on only the study group with passive diffusion badges (67). Blood samples were drawn and analyzed for PCE concentration before the work week (67). The median time working on the day of badge-wearing was 5 hours and 45 min, and the median range worked the week prior was 3.25–8 hours (67). Clinical symptoms were assessed for the exposed and control group via medical examination and questionnaires. The overall mean for personal air sampling was 47.41 mg/m^³^, and the recorded range was 1.5–221 mg/m^³^ (67). The majority of recorded personal air sampling measurements were within the established OSHA and ACGIH OELs. Blood samples were analyzed on 49/50 subjects (67). The average recorded PCE concentration in blood sampling was 125.9 μg/L (0.1259 mg/L), with a range of 11.8–544 μg/L (0.0118–0.544 mg/L) (67). Of all workers, eight percent had PCE levels higher than 400 μg/L (0.400 mg/L), falling either close to or outside of the established ACGIH BEI for blood levels of .05mg/L, drawn before shift (67, 68). Working time and personal sampling levels were not correlated with reported clinical symptoms; however, of the recorded clinical symptoms, 78% of the exposed employees reported one symptom possibly related to PCE exposure, mainly neurological (87%) (67).

Modenese et al. conducted personal air sampling as well as biological sampling via exhaled air measurements and urine concentration measurements. The study population included 21 dry cleaning shops and 60 workers (69). Personal passive samplers were placed on each worker for an entire eight-hour work shift, and alveolar air and urine samples were collected at the end of the work shift (69). Results of personal air sampling showed a mean concentration of 17.0 mg/m^³^ (SD: 18.5 mg/m^³^) and a range of 0.1–86.0 mg/m^³^ within the established OSHA and ACGIH OEL (69). As for biological sampling measurements, the mean exhaled alveolar air concentration was 10.4 mg/m^³^ (SD: 10.3 mg/m^³^), with a range of 0.1–32.4 mg/m^³^ (69). While mean urine samples measured 8.4 μg/L (0.0084 mg/L) (SD:11.7 μg/L or 0.0117 mg/L) with a range of 0.1–40.0 μg/L (0.0001–0.04 mg/L) (69). Only an established ACGIH BEI is available for exhaled air collected before the start of the shift, 3ppm (20.34 mg/m^³^) (68, 69). Most exhaled air samples were within the range of established BEI; however, a few measurements exceeded the set value (69). Although Modenese et al. collected alveolar air post-shift, and the typical procedure by ACGIH is to collect exhaled air prior to the shift, it still provides a quantitative comparison and exposure insight.

Dias et al. conducted personal and biomonitoring exhaled air sampling among 25 individuals in 24 dry cleaning facilities. Additional sampling was taken in an electroplating facility, research laboratory, and automotive paint preparation shop (70). All personal air samples collected from the 24 dry cleaning facilities, except facility 10, had PCE concentrations exceeding the inhalation reference concentration (IRC) recommended by the EPA of 0.016 mg/m^³^; however, it is important to note that the concentrations did not exceed OSHA or ACGIH OEL standards (70). The study only reported the combined sampling data, including the three sample sites outside the specified occupation. Nevertheless, the paper states that the highest concentrations of PCE belonged to those samples collected within the dry cleaning facilities and from dry cleaning workers (70). Personal sampling results ranged from 14.0–3,205 μg/m^³^ (0.014–3.205 mg/m^³^) with a median concentration of 599.0 μg/m^³^ (0.599 mg/m^³^) (70). Exhaled air of exposed individuals had concentrations ranging from 6.0–2,635 μg/m^³^ (0.006–2.635 mg/m^³^) with a median concentration of 325 μg/m^³^ (0.325 mg/m^³^) within ACGIH BEI for exhaled air (70). Associated health impacts were not measured or monitored throughout this study.

Azimi et al. conducted strictly biological sampling, examining peripheral blood via comet assay (61). The study population included 33 dry cleaners and 26 matched non-exposed individuals (61). Samples were collected from each participant in the morning, and the comet assay was performed following the Singh et al. protocol, with slight modifications (61, 71). Fifty cells were counted on each comet slide and assessed by comet assay parameters (TL, %DNA in tail, TM, and olive TM). Results found a significant increase in early DNA damage among the exposed individuals vs. the non-exposed, as primary DNA damage to leukocytes in dry cleaners was high (exposed median tail length: 25.85 vs. non-exposed: 5.61; exposed median %DNA in tail: 23.03 vs. non-exposed: 8.77; exposed median tail moment: 7.07 vs. non-exposed: 1.03) (61). However, the duration of employment in the dry cleaning industry was not correlated with DNA damage. This could be attributed to the fact that comet assay analysis on blood lymphocytes only reflects recent exposure to DNA damage, which is typically repairable (61).

Ziener and Braunsdorf collected biological sampling via end-exhaled breath in a field study conducted in one dry cleaning shop on four workers and a control group of 10 subjects (72). Samples were collected one day before the working shift, twice consecutively (72). PCE concentrations in the exposed group ranged from 3.4–16.7 μg/L (3.4–16.7 mg/m^³^), with a mean of 9.35 μg/L (9.35 mg/m^³^), within the established ACGIH BEI for in end exhaled air (20.34 mg/m^³^) (68, 72). No health outcome assessment information was collected during this study.

#### 5.2.2. TCE

Two studies, Friesen et al. and Sadeghi et al., measured TCE concentration in China and Iran. Friesen et al. used the Shanghai Database of Inspection Measurements, which did not report the number of dry cleaning facilities or workers involved. Sadeghi et al. only reported the number of dry cleaning facilities; ten. Both studies collected ambient area air samples in the dry cleaning occupational setting. Further details regarding the reported TCE concentrations and study characteristics are presented in Table 1.

Friesen et al. conducted a retrospective survey study examining short-term area air sampling collected between 1968–2000 among various industries and occupations, including the laundry and dry cleaning industry (73). The database included the sampling date, industry names, location of the sampling device, and air concentration (73). However, the database did not note the sampling and analytical methods used to evaluate TCE, a study limitation. Additionally, the study did not monitor health impacts or outcomes from TCE exposure. Industry-specific differences in air concentration measurements were analyzed and compared (73). The database presented 932 TCE measurements sampled (73). Twenty-three laundry and dry cleaning industry samples were collected between 1976–1977. Of those samples, the arithmetic mean was 710 mg/m^³^, the geometric mean was 570 mg/m^³^, the geomatic standard deviation was 2.0 mg/m^³^, and the maximum recorded measurement was 2,200 mg/m^³^ (73). All area measurements taken within the laundry and dry cleaning facilities measured far greater than the established OEL for TCE (OSHA: PEL 8-hour TWA: 100 ppm (535 mg/m^³^) ACGIH: TLV 8-hour TWA: 10 ppm (54 mg/m^³^) NIOSH: REL 10-hour TWA: 25 ppm (134.3 mg/m^³^)) (22, 24, 26). However, it is essential to consider that these measurements were taken in the late 1970s, likely before implemented regulations. Further, based on the conducted mixed-effects model, the paper concludes that TCE air concentrations have declined between the specified period (73).

Sadeghi et al., in addition to measuring PCE samples previously discussed, also sampled for TCE. Of the ten dry cleaning shops assessed, mean TCE concentration in area air ranged from 29.5–543.7 μg/L (29.5–543.7 mg/m^³^), with a grand mean of 95.69 μg/L (95.69 mg/m^³^), and a maximum measurement of 964 μg/L (964 mg/m^³^) (66). The range of means for TCE varied greatly, with specific samples measuring within and far outside the acceptable range (66). The grand mean is within OSHAs and NIOSHs OELs, but outside of ACGIHs OEL, and the maximum recorded value also fell far outside of all established OELs (OSHA: PEL 8-hour TWA: 100 ppm (535 mg/m^³^) ACGIH: TLV 8-hour TWA: 10 ppm (54 mg/m^³^) NIOSH: REL 10-hour TWA: 25 ppm (134.3 mg/m^³^)) (22, 24, 26, 66).

#### 5.2.3. TCA

Modenese et al., in addition to measuring PCE concentrations in personal air and two types of biological sampling previously discussed, also measured TCA concentrations via urine sampling (69). The mean TCA concentration measured among 60 workers from 21 varying shops was 0.7 mg/L (SD: 0.9 mg/L), with a median concentration of 0.3 mg/L and a range of 0.02–3.2 mg/L (69). ACGIH BEI for TCA measured at the end of a shift at the end of the workweek is 15 mg/L (69). All measurements collected were within the established ACGIH BEI for TCA of 15 mg/L measured at the end of a shift at the end of the workweek (68). No health outcomes were measured.

#### 5.2.4. Benzene

Benzene was examined in only one study, a case report conducted by Shim et al. in South Korea, examining two elderly individuals, one male and one female, who had worked together in a small dry cleaning shop (40m^²^) for 40 years (63). Throughout that time, they had been exposed to dry cleaning solvents, specifically benzene (63). The 60-year-old man without a significant medical history was admitted to the hospital with jaundice and was later identified to have a mass in his abdomen (63). Biological samples were collected, and laboratory findings returned abnormal (total/direct bilirubin: 18.4/9.9 mg/dL; AST/ALT 183/331 IU/L; ALP 700 IU/L; GGT 537 IU/L; CA19–9 of 4,980 U/mL) (63). The patient was diagnosed with stage IV gallbladder cancer. The female patient, a 60-year-old female, was similarly admitted to the hospital for jaundice, and her laboratory findings also came back abnormal (total/direct bilirubin: 9.8/6.4 mg/dL; AST/ALT 172/497 IU/L; ALP 411 IU/L; GGT 1,304 IU/L; CA19–9 of 613 U/mL) (63). Like the male, the female patient was diagnosed with metastasized gallbladder cancer (63). Neither of the patients had an elevated risk factor for gallbladder cancer compared to the general population (63).

Both patients had urinary phenol and t,t-muconic acid testing conducted, metabolites of benzene, to determine their internal benzene concentrations (63, 74). The male patient’s urine phenol-benzene measured 12.895 mg/g creatine (128.95 μg/g), while the female was 2.489 mg/g creatine (24.89 μg/g) (63). The male’s urine t,t-muconic acid-benzene measured 0.057 mg/g creatine (0.570 μg/g), while the females measured 0.058 mg/g creatine (0.580 μg/g) (63). According to Shim et al., both patients’ benzene levels were measured within the normal range for urine phenol-benzene (< 50 mg/g creatine, ten ppm standard) and t,t-muconic acid-benzene (< 1mg/g creatine, ten ppm standard) (63). Furthermore, both patients did not exceed the established ACGIH BEI for t,t-muconic acid in urine for benzene collected at the end of the shift of 500 μg/g creatinine (68). A likely explanation could be that benzene is broken down by the body over time, with a biological half-life of approximately 24 hours (63).

#### 5.2.5. Butylal and High-Flashpoint Hydrocarbon

Butylal and high-flashpoint hydrocarbons were examined in one paper via personal and area sampling conducted by Ceballos et al. (28). In four shops, two shops had samples collected for butylal and its hydrolysis by-products, formaldehyde, and butanol (28). The remaining shops had samples collected for the high-flashpoint hydrocarbon DF-2000 (28). The total number of workers was not identified. Full-shift and task-based personal samples and full-shift and short-term area air samples were taken in all shops (28). The study found that full-shift personal exposure levels to DF-2000 ranged from 0.99–5.4 mg/m^³^, while full-shift personal exposure to butylal ranged from 0.017–0.83 ppm (0.1114–5.440 mg/m^³^) (28). Task-based personal exposure levels were higher for both DF-200 and butylal, ranging from < 3.8 mg/m^³^−7.9 mg/m^³^ and 0.42–1.9 ppm (2.753–12.45 mg/m^³^), respectively (28). The greatest task-based exposures were observed near the dry cleaning machines or during the fabric pressing process, with butylal and DF-200 levels reaching 0.83 ppm (5.442 mg/m^³^) and 5.4 mg/m^³^, respectively, (28). Formaldehyde was detected in one full-shift personal sample at 0.0087 ppm, while butanol was < 0.001 ppm (28). Area sampling revealed full-shift concentrations of 0.16–5.6 mg/m^³^ for DF-2000 and 0.0039–0.31 ppm (0.0255–2.03 mg/m^³^) for butylal (28). Short-term area samples ranged from 5.3–37.0 mg/m^³^ for DF-2000 and 0.17–1.9 ppm (1.114–12.46 mg/m^³^) for butylal (28). No health outcome measurements were monitored in this study, and there are currently no available OELs from OSHA, ACGIH, or NIOSH to compare.

#### 5.2.6. Various VOCs

One study (Eun et al.) examined various VOCs using area air sampling from one laundry facility in South Korea (75). The number of workers in the facility was not disclosed. The sampling was performed thrice during a 23-minute dry-cleaning process (75). There were 77 analytes examined (75). Photochemical ozone creation penitential (POCP) was estimated via a method proposed by Derwent et al. (75, 76). The secondary organic aerosol formation potential (SOAP) was estimated by multiplying the emissions by the degree to which the compound produces SOA in the presence of additional mass concentration relative to the SOA formed when the same amount is present (75). This study additionally conducted a risk assessment following the National Research Council procedures, including hazard identification and dose-response assessments for carcinogenic and non-carcinogenic compounds (75, 77).

Results showed that 61% of the 77 substances monitored during the dry-cleaning process were detected, with nonane, decane, undecane, nonanal, and decanal emitted the most (75). Of those substances, there were 19 chemicals to which the POCP equation could be applied (total = 33.7 ppm. nonane 41.3% (74.28 mg/m^³^); decane 34.2% (68.33 mg/m^³^); undecane 13.6% (29.80 mg/m^³^); o-xylene 5.5% (8.185 mg/m^³^) = 95% of ozone creation) (75). There were 18 chemicals to which the SOAP calculations could be applied (total = 8.3 ppm. xylene 27.5% (10.08 mg/m^³^); decane 27.2% (13.36 mg/m^³^); undecane 25% (13.49 mg/m^³^); nonane 9.4% (4.163 mg/m^³^); Toluene 3.2% (1.018 mg/m^³^)) (75). Despite the relatively short total sampling duration of 69 minutes, comparing the established exposure limits can help identify areas that may require further implementation of control measures. Of the identified substances, OELs are only available for nonane (ACGIH: TLV 8-hour TWA: 200ppm (1049 mg/m^3^); NIOSH: REL 10-hour TWA: 200ppm (1049 mg/m^3^)), o-xylene (OSHA: PEL 8-hour TWA: 100 ppm (435 mg/m^³^) ACGIH: TLV 8-hour TWA: 20 ppm (86.83 mg/m^³^) NIOSH: REL 10-hour TWA: 100 ppm (435 mg/m^³^)), and toluene (OSHA: PEL 8-hour TWA: 200 ppm (750 mg/m^³^) ACGIH: TLV 8-hour TWA: 20 ppm (75.33 mg/m^³^) NIOSH: REL 10-hour TWA: 100 ppm (375 mg/m^³^)) (78, 79, 80, 81). All values were within the OELs.

The observed carcinogens had a mean total estimated cancer risk of 2.36 × 10^−5^, nitrobenzene having the highest cancer risk (1.26 × 10^−4^), and acrylonitrile, carbon tetrachloride, nitrobenzene, bromodichloromethane, and chloromethane exceeding standards (75). Of the 11 non-carcinogenic substances, the mean total hazard quotient was 1.19, with bromomethane having the highest risk index at 5.95, and bromomethane, chlorobenzene, o-xylene, and heptachlor-1,3-butadiene exceeding standards (75). It is essential to acknowledge that the study had limitations since it relied on a single machine’s measurements to estimate the emission concentration.

## 6. Discussion

Despite employing a broad search criterion to encompass all relevant dry cleaning solvent types, the predominant focus of the published papers within the last decade pertained to PCE, despite the industry’s ongoing transition towards safer alternatives and reduced reliance on the substance. This highlights a notable discrepancy between the dry cleaning industry’s trajectory and the current occupational exposure research focus. Further, studies examining PCE concentrations in the dry cleaning industry presented exposure risks with area air sampling studies revealing that certain working positions, such as unloading clothes from the dry-cleaning machines, demonstrated notably higher levels of PCE (65). However, the lack of associated health outcome measurements in these studies limits our understanding of the actual health implications of such exposures. On the other hand, studies that collected biological samples demonstrated genotoxic effects amongst dry cleaners, including increased MN frequency and DNA damage (62). These effects were observed even at PCE concentrations below the established occupational exposure limits, suggesting that current limits may not adequately protect against genotoxic damage and that more stringent OELs may benefit workers’ health.

The two studies on TCE and the one examining Benzene exposure provided exposure-related insights. Friesen et al.’s retrospective survey revealed high TCE concentrations in the late 1970s, surpassing established exposure limits. However, these measurements were conducted before current OEL regulations were in place. In Sadeghi et al.’s more recent study, TCE concentrations varied, with the grand mean falling within acceptable limits but sporadic instances of high exposure. Although neither study explored the health effects of TCE exposure, TCE, like benzene, is classified as a group 1 carcinogen, indicating its genotoxicity based on substantial human data, and therefore, that there is no safe level of exposure (5, 82). Shim et al.’s study did, however, provide health-related data, reaffirming benzene exposure risks. Governments globally are moving to ban cancer-linked chemicals like benzene and TCE from the dry cleaning industry. As a result, usage is expected to decline over time. However, monitoring is still necessary for those facilities that still use these substances to ensure exposures do not surpass OEL; even sporadic instances of high exposure can have detrimental health effects, highlighting the ongoing need for precautionary measures.

The available literature on solvents used in the laundry and dry cleaning industry reveals a significant gap in research pertaining to alternative solvents to PCE. Among the three published studies (Modenese et al., Ceballos et al., & Eun et al.) exploring less studied but still widely used solvents TCA, butylal, high-flashpoint hydrocarbons, and one study examining various VOCs (nonane, decane, undecane, xylenes, & toluene), all study designs failed to monitor exposure related health outcomes. This lack of data regarding the health effects of alternative solvents is alarming, especially considering the limited availability of OEL for these substances. Currently, only nonane, o-xylene, and toluene have established OELs, and TCA has a biological exposure index (BEI) for urinary levels, of which all samples fell within standard ranges (68, 78, 79, 80).

The examination of human health effects is of the utmost importance in studies investigating newly introduced solvents, and the scarcity of this type of published literature plays a significant role in the absence of established OELs for these solvents. Further, this present limitation has additionally contributed to the lack of IARC carcinogenicity classification for these solvents, with only TCA, xylenes, and toluene having been classified as Group 2B (possibly carcinogenic) and Group 3 (uncertain carcinogenicity) by IARC, respectively (83, 84, 85). Consequently, dry cleaning workers face exposure to solvents that pose unknown health risks without clear information regarding exposure to carcinogenicity or specific types of associated cancers. Compounding this issue is the unavailability of accessible information about the effects of exposure to these solvents. The resources expected to provide insights, such as agencies responsible for establishing OELs and evaluating carcinogenicity levels, lack the necessary data to inform workers about these critical concerns.

## 7. Conclusion

The present scoping review provides insight into occupational exposures in the dry cleaning industry by comparing study area, personal, and biological measurements to existing OELs, as well as an assessment of substance IARC classification. Despite significant diversity across study methodologies, most (66%) included studies failed to investigate exposure-related health outcomes. Furthermore, all studies investigating emerging dry cleaning solvents (alternatives to PCE), which presently lack human carcinogenicity data, did not assess any health-related outcomes. These findings underscore a significant gap in our understanding of the potential risks associated with these solvents. Results indicate a lack of published data examining carcinogenic effects and specific cancer associated with particular solvents. Additionally, studies that assessed PCE exposure levels and corresponding health outcomes have revealed a potential association between exposure and DNA damage, even at exposure levels below OELs. These findings emphasize a need for additional investigations to determine safer OEL values for PCE.

Future studies should prioritize investigating the potential health effects and carcinogenic properties of exposure to new and alternative solvents, such as butylal, and high-flashpoint hydrocarbons, two substances that lack OEL and IARC classification. Transformation and genotoxicity assay studies are crucial first steps that need to be taken to assess the potential carcinogenicity of exposure to these emerging and supposedly greener alternative chemicals in the dry cleaning industry. These in vitro studies will help determine if there is a causal relationship between solvent exposure and cancer development as new solvents become more widely used. In addition, large-scale observational studies that monitor exposure levels and health effects should be conducted at facilities already utilizing alternative solvents to PCE to close the current research gap.

While this scoping review primarily aims to identify gaps in the existing literature concerning exposure risks and associated health outcomes in the dry cleaning industry, the implications of these findings extend beyond this specific industry to other industries and the general population engaging with solvents. Most individuals conduct laundry activities and may inadvertently expose themselves to harmful solvents commonly used in dry cleaning. Therefore, the recommendations derived from this review can benefit those working in the dry cleaning industry and potentially safeguard the general public’s health.

## 8. SI

The supplementary Information document contains VOC background information and tables regarding the used inclusion and exclusion criteria (SI Table S1), search terms (SI Table S2), articles returned for search by database (SI Table S3), and the final retrieved scoping review articles (SI Table S4).

## Figures and Tables

**Table 1.** Summary of studies investigating area air sampling concentrations of PCE, butylal, Df-2000, TCE, and various VOCs

Reference	Country	Number of workplace and workers	Exposure Measurement Conditions	Exposure Agent of Interest	Measured Outcomes (mg/m^3^)	Available Reference OEL
**Area Air Sampling**						
Ceballos et al. (28)	USA	4 dry cleaning shops/not reported	Personal and Area Sampling: At least two days evaluating each shop. Full-shift and short-term area air sampling. Duration of machine dry cleaning 70–80 min. No temp, reported.	Butylal and high-flashpoint hydrocarbons (Df-2000)	**Df-2000 full shift range:** 0.1600–5.600 **Df-2000 short-term range:** 5.300–37.00 **Butylal full shift range:** 0.02557–2.032* Butylal short-term range: 1.115–12.46*	**NA**
Eun et al. (75)	South Korea	1 laundry facility/not reported	Area Sampling: 1 kg of cotton fiber washed with petroleum solvent. Experiment performed 3x. Duration of machine dry cleaning 23 min. Avg. temp 20°C at 1 atm.	VOCs	**Photochemical Ozone Creation Potential:** Total: 33.7ppm Nonane: 74.28* Decane: 68.33* Undecane: 29.80* O-xylene: 8.185* Secondary Organic Aerosol Formation: Total: 8.3ppm Xylene: 10.08* Decane: 13.36* Undecane: 13.49* Nonane: 4.163* Toluene: 1.018*	**Nonane:** **ACGIH:** TLV-TWA (8hr.) 200ppm (1050 mg/m^3^) (78) **NIOSH:** REL-TWA (1 Ohr.) 200ppm (1050 mg/m^3^) (78) **O-xylene:** **OSHA:** PEL-TWA (8hr.) 100ppm (435 mg/m^3^) (79) **ACGIH:** TLV-TWA (8hr.) 20ppm (86.83 mg/m^3^) (81) **NIOSH:** REL-TWA (1 Ohr.) 100ppm (435 mg/m^3^) REL-STEL (15min.) 150ppm (655 mg/m^3^) (79) **Toluene:** **OSHA:** PEL-TWA (8hr.) 200ppm (750 mg/m^3^) (80) **ACGIH:** TLV TWA (8hr.) 20ppm (75.33 mg/m^3^) (80) **NIOSH:** REL-TWA (1 Ohr.) 100ppm (375 mg/m^3^) REL-STEL (15min.) 150ppm (560 mg/m^3^) (80)
Friesen et al. (73)	China	Not reported/not reported	Area Sampling: Database measurments recorded 932 short-term (≤20min.) measurments collected across different occupations. 23 samples collected in the laundry industry. Limited data on sample and analytic method. Temp. at 25°C at 1 atm.	TCE	**Short-term area exposure concentrations laundiy and diy cleaning only:** AM: 710.0 GM: 570.0 GSD: 2.000 Max: 2,200	**OSHA:** PEL-TWA (8hr.) 100ppm (535 mg/m^3^) (22) **ACGIH:** TLV-TWA (8hr.) 10ppm (54 mg/m^3^) (22) TLV-STEL (15min.) 25ppm (135 mg/m^3^) **NIOSH:** REL-TWA (10hr.) 25ppm (134.3 mg/m^3^) (22)
Habib et al. (65)	UAE	4 dry cleaning shops/not reported	Area Sampling: Air sampler set up at three positions in each facility. Samples were collected for three 8 hr. workdays. No temp. reported.	PCE	**Facility A range:** 52.28–1,356* ► **Facility B range:** 47.47–508.7* ► **Facility C range:** 20.34–3,458* ► **Facility D range:** 7.798–1,627* ►	**OSHA:** PEL-TWA (8hr.) 100ppm (678 mg/m^3^) (21) **ACGIH:** TLV-TWA (8hr.) 25ppm (170 mg/m^3^) (21) TLV-STEL (15min.) 100ppm (685 mg/m^3^) **NIOSH:** Lowest feasible concentration (21)
Sadeghi et al. (66)	Iran	10 dry cleaning shops/not reported	Area Sampling: Four samples taken at each dry cleaning shop - one sample every 15 days. No temp. reported.	PCE and TCE	**PCE:** **Grand mean:** 110.9* Mean range: 42.70–516.0* ► **Max:** 960.0* Min: 22.00* TCE: **Grand** **mean:** 95.69* Mean range: 29.50–543.7* ► **Max:** 964.0* Min: 25.00*	**PCE:** **OSHA:** PEL-TWA (8hr.) 100ppm (678 mg/m^3^) (21) **ACGIH:** TLV-TWA (8hr.) 25ppm (170 mg/m^3^) (21) TLV-STEL (15min.) 100ppm (685 mg/m^3^) **NIOSH:** Lowest feasible concentration (21) **TCE:** **OSHA:** PEL-TWA (8hr.) 100ppm (535 mg/m^3^) (22) **ACGIH:** TLV-TWA (8hr.) 10ppm (54 mg/m^3^) (22) TLV-STEL (15min.) 25ppm (135 mg/m^3^) **NIOSH:** REL-TWA (10hr.) 25ppm (134.3 mg/m^3^) (22)

SD= Standard deviation; AM= Arithmetic mean; GM=Geometric mean; GSD= Geometric standard deviation

* = Concentrations were reported in a different unit and calculated in mg/m^3^ as given in the methods section; = Above at least one of the presented OEL values

► = Maximum range value outside at least one of the presented OEL values

NA = Not available

**Table 2:** Summary of studies investigating personal air sampling concentrations of PCE, butylal, and Df-2000

Reference	Country	Number of workplace and workers	Exposure Measurement Conditions	Exposure Agent of Interest	Measured Outcomes (mg/m^3^)	Available Reference OEL
**Personal Air Sampling**						
Ceballos et al. (28)	USA	Four dry cleaning shops/not reported	Personal and Area Sampling: At least two days evaluating each shop. Full-shift and task-based personal samples collected. Duration of machine dry cleaning 70–80 min. No temp. reported.	Butylal and high-flashpoint hydrocarbons (Df-2000)	**Df-2000 full-shift range:**0.990–5.400 **Df-2000 task-based range:** <3.800–7.900 **Butylal full-shiftrange:** 0.1114–5.440* Butylal task-based range: 2.753–12.45*	**NA**
Dias et al. (70)	Brazil	24 dry cleaning shops, one electroplating industry, one research laboratory, one automotive paint shop/25 workers	Personal and Biological Sampling: Samples collected at the end of 8 hr. work shift in breathing zone of workers for 15 min. post collection of exhaled air samples. Mean indoor temp. 24 ± 3°C.	PCE	**Median:** 0.599* Range: 0.014–3.205*	**OSHA:** PEL TWA (8hr.) 100ppm (678 mg/m^3^) (21) **ACGIH:** TLV-TWA (8hr.) 25ppm (170 mg/m^3^) (21) TLV-STEL (15min.) 100ppm (685 mg/m^3^) **NIOSH:** Lowest feasible concentration (21)
Everatt et al. (62)	Lithuania	Six dry cleaning shops/59 volunteers (30 exposed and 29 non-exposed)	Personal and Biological Sampling: Breathing zone samples collected on two consecutive workdays in 150-min. intervals post 8hr. shifts. Avg. temp. 23°C.	PCE	**Mean (SD):** 31.40 (23.51) **Range:** 0.0–77.46	**OSHA:** PEL TWA (8hr.) 100ppm (678 mg/m^3^) (21) **ACGIH:** TLV-TWA (8hr.) 25ppm (170 mg/m^3^) (21) TLV-STEL (15min.) 100ppm (685 mg/m^3^) **NIOSH:** Lowest feasible concentration (21)
Lucas et al. (67)	France	22 dry cleaning shops/145 volunteers (50 exposed and 95 non-exposed)	Personal and Biological Sampling: Personal passive samplers placed for a full work-shift on a single day. No temp. reported.	PCE	**Mean:** 47.41 **Median:** 25.5 **Range:** 1.5–221 ►	**OSHA:** PEL TWA (8hr.) 100ppm (678 mg/m^3^) (21) **ACGIH:** TLV-TWA (8hr.) 25ppm (170 mg/m^3^) (21) TLV-STEL (15min.) 100ppm (685 mg/m^3^) **NIOSH:** Lowest feasible concentration (21)
Modenese et al. (69)	Italy	21 dry cleaning shops/60 workers	Personal and Biological Sampling: Personal passive samplers worn for a full 8hr. Work-shift on a single day. No temp. reported.	PCE	**Mean (SD):** 17 0 (18.5) **Median:** 10.6 **Range:** 0.1–86.0	**OSHA:** PEL TWA (8hr.) 100ppm (678 mg/m^3^) (21) **ACGIH:** TLV-TWA (8hr.) 25ppm (170 mg/m^3^) (21) TLV-STEL (15min.) 100ppm (685 mg/m^3^) **NIOSH:** Lowest feasible concentration (21)

SD = Standard deviation; AM = Arithmetic mean; GM = Geometric mean; GSD = Geometric standard deviation

* = Concentrations were reported in a different unit and calculated in mg/m^3^ as given in the methods section; = Above at least one of the presented OEL values

► = Maximum range value outside at least one of the presented OEL values

NA= Not available

**Table 3:** Summary of studies investigating biological sampling concentrations of Benzene, PCE, and TCA

Reference	Country	Number of workplace and workers	Exposure Measurement Conditions	Exposure Agent of Interest	Measured Outcomes (mg/m^3^)	Available Reference OEL
**Biological Sampling**						
Azimietal. (61)	Iran	Not reported/59 volunteers (33 exposed and 26 non-exposed)	Biological Sampling (Peripheral Blood and Comet Assay): Peripheral blood samples collected in the morning from each participant. Cells counted in each comet slide and analyzed.	PCE	**Comet Assay Exposed Group:** **Tail length range** 6.63–67.2μm **Median:** 25.85μm **DNA% in tail range:** 5.73–48.85 **Median:** 23.03 **Tail moment range** 0.42–44.29 **Median:** 7.07	**ACGIH BEI in blood:** samples collected prior to start of shift 0.5 mg/L (68)
Dias et al. (70)	Brazil	24 dry cleaning shops, one electroplating industiy, one research laboratory, one automotive paint shop/25 workers	Personal and Biological Sampling (Exhaled Air): Post 8hr. work-shift workers exhaled air for 30 sec into Teflon tube. Mean indoor temp. 24 ± 3°C.	PCE	**Median:** 0.325* Range: 0.006–2.635*	**ACGIH BEI in end exhaled air** samples collected prior to start of shift 3ppm (20.34 mg/m^3^) (68)
Everatt et al. (62)	Lithuania	Six dry cleaning shops/59 volunteers (30 exposed and 29 non-exposed)	Personal and Biological Sampling (Peripheral Blood and Comet Assay): Venous blood from exposed and control subjects, and comet assay in peripheral blood lymphocyte collected. Chromosome aberrations (CAs), micronuclei (MN), and DNA damage via comet assay analyzed.	PCE	**Exposed Group:** **Comet Assay** **CA frequency (CA/100cell) significant findings:** **Total CA mean (SD):** 3.15(1.81) **Total chromosome-type aberrations (SD):** 1.04 (0.66) **Total chromatid-type aberrations (SD):** 2.11 (1.67) **Chromatid breaks (SD):** 2.03 (1.68) **MN frequency (CN/1000 Dinudeated cells) significant findings:** **Total MN mean (SD):** 11.36(6.90) **Comet mean tail length in Dlood lymphocytes:** **Total tail length mean (SD):** 10.45 (6.52) μm	**ACGIH BEI in blood:** samples collected prior to start of shift 0.5 mg/L (68)
Lucas et al. (67)	France	22 dry cleaning shops/145 volunteers (50 exposed and 95 non-exposed)	Personal and Biological Sampling (Peripheral Blood): Blood levels for PCE drawn at the beginning of workday. Samples analyzed on 49/50 exposed subjects).	PCE	**Mean PCE blood level:** 125.9 μg/l **Median:** 73.6 μg/l **Range:** 11.8–544 μg/l	**ACGIH BEI in blood:** samples collected prior to start of shift 0.5 mg/L (68)
Modenese et al. (69)	Italy	21 dry cleaning shops/60 workers	Personal and Biological Sampling (Exhaled Air and Urine): Samples of exhaled alveolar air collected to examine PCE. Urine samples collected and analyzed for PCE and trichloroacetic acid (TCA). Samples obtained a the end of 8hr. work-shift.	PCE and TCA	**Exhaled Air PCE:** **Mean (SD):** 10.4 (10.3) **Median:** 6.6 **Range:** 0.1–37.4 ► **Urine:** **PCE:** **Mean (SD):** 8.4 μg/l (11.7) **Median:** 3.1μg/l **Range:** 0.1–40.0 μg/l **TCA** **Mean (SD):** 0.7 μg/l (0.9) **Median:** 0.3 μg/l **Range:** 0.02–3.2 pg/l	**ACGIH BEI PCE in end exhaled air:** samples collected prior to start of shift 3ppm (20.34 mg/m^3^) (68) **ACGIH BEI TCA in urine:** Samples collected at end of shift 15mg/L (68)
Shim et al. (63)	South Korea	One dry cleaning shop/2 workers	Biological Sampling (Urine and Blood): After showing symptoms of jaundice, subjects were admitted to the hospital, where blood and urine samples were taken for laboratory work.	Benzene	**Subject A** **Urine phenol-benzene:** 2.489 mg/g creatinine **t.t-munoic add-benzene:** 0.058 mg/g creatinine **Subject B:** **Urine phenol-benzene:** 12.895 mg/g creatinine **t.t-munoic** **add-benzene:** 0.057 mg/g creatinine	**ACGIH BEI t,t-Muconic Acid in urine:** Samples collected at end of shift 500 μg/g creatinine (68)
Ziener & Braunsdorf (72)	Germany	One dry cleaning shop/14 volunteers (4 exposed and 10 non-exposed)	Biological Sampling (Exhaled Air): End-exhaled air sampling collected a week prior to last shift of working week. Two samples were collected consecutively. Subjects filled sampling tubes for approx. 4 min. Avg. temp. 20°C.	PCE	**Mean (exposed):** 9.35* Range (exposed): 3.4–16.7*	**ACGIH BEI in end exhaled air:** samples collected prior to start of shift 3ppm (20.34 mg/m^3^) (68)

SD = Standard deviation; AM = Arithmetic mean; GM = Geometric mean; GSD = Geometric standard deviation

* = Concentrations were reported in a different unit and calculated in mg/m^3^ as given in the methods section; = Above at least one of the presented OEL values

► = Maximum range value outside at least one of the presented OEL values

NA = Not available

**Table 4:** Reported health outcomes and IARC classification by exposure type

Reference	Study Design	Reported Health Impact in Paper		IARC Classification
**PCE**				
Azimi et al. (61)	Case Control Study	Greater DNA damage in dry cleaners exposed to PCE compared to non-exposed. Evidence of genotoxic and carcinogenic effects.		Group 2A- Probably carcinogenic to humans (5)
Dias et al. (70)	Cross Sectional and Biomonitoring Study	No health impacts monitored		
Everatt et al. (62)	Case Control Study	Dry cleaning workers showed a significant increase in micronuclei and DNA damage compared to controls. No significant difference in CA. The frequency of exposure and employment duration was associated with CA frequency.		
Habib et al. (65)	Cross Sectional Study	No health impacts monitored.		
Lucas et al. (67)	Cross Sectional Study	No increased rate of clinical symptoms or acute exposure symptoms related to PCE.		
Modenese et al. (69)	Cross Sectional and Biomonitoring Study	No health impacts monitored.		
Sadeghi et al. (66)	Cross Sectional Study	No health impacts monitored.		
Ziener & Braunsdorf. (72)	Case Control and Biomonitoring Study	No health impacts monitored.		
**TCE**				
Friesen et al. (73)	Retrospective Survey Study	No health impacts monitored.		Group 1 - Carcinogenic to humans (5)
Sadeghi et al. (66)	Cross Sectional Study	No health impacts monitored.		
**TCA**				
Modenese et al. (69)	Cross Sectional and Biomonitoring Study	No health impacts monitored.		Group 2B - Possibly carcinogenic to humans (83)
**Benzene**				
Shim et al. (63)	Case Report Study	Jaundice and subsequent diagnosis of late-stage gallbladder cancer.		Group 1 - Carcinogenic to humans (82)
**Butylal and high-flashpoint hydrocarbons**				
Ceballos et al. (28)	Cross Sectional Study	No health impacts monitored.		NA
**Various other VOCS**				
		**Type of VOC**	**Reported Health Impact in Paper**	
Eun et al. (75)	Cross Sectional Study	Nonane	No health impacts monitored.	NA
		Decane		NA
		Undecane		NA
		Xylenes		Group 3 -Unclassifiable as to carcinogenicity in humans (84)
		Toluene		Group 3 -Unclassifiable as to carcinogenicity in humans (85)

CA = Chromosome Abrasion; NA = Not available

## Abbreviations

ILO: International Labour Organization
VOC: Volatile organic compounds
OEL: Occupational exposure limits
TCE: Trichloroethylene
PCE: Perchloroethylene
SMR: Standardized Mortality Ratio
NIOSH: National Institute for Occupational Safety and Health
SMR: Standardized mortality ratio
CI: Confidence interval
IARC: International Agency for Research on Cancer
OSHA: Occupational Safety and Health Administration
PEL: Permissible exposure limit
ppm: Parts per million
TWA: Time-weighted average
ACGIH: American Conference of Governmental Industrial Hygienists
TLV: Threshold limit value
STEL: Short-term exposure limit REL: Recommended exposure limit
EPA: Environmental Protection Agency
REACH: Registration, Evaluation, Authorization, and Restriction of Chemicals
NICNAS: Australia’s National Industrial Chemicals Notification and Assessment Scheme
TURI: Toxic Use Reduction Institute
HAPs: Hazardous air pollutants
ODS: Ozone-depleting substances
PGE: Propylene Glycol Ethers
DPnB: Glycol n-butyl
DPtB: Dipropylene glycol tert-butyl
n-PB: n-Propyl Bromide
REACH: Registration, Evaluation, Authorization, and Restriction of Chemicals
PRISMA-ScR: Preferred Reporting Items for Systematic Reviews and Meta-Analyses- Scoping Reviews
BEI: Biological Exposure Indices
TL: Tail length
TM: Tail moment
CA: Chromosome aberration
MN: Micronuclei
TCA: Trichloroacetic acid
IRC: Inhalation reference concentration
SOAP: Secondary organic aerosol potential
POCP: Photochemical ozone creation penitential
SD: Standard deviation
GM: Geometric mean
GSD: Geometric standard deviation
